# Pyridine 2,4-Dicarboxylic Acid Suppresses Tomato Seedling Growth

**DOI:** 10.3389/fchem.2018.00003

**Published:** 2018-01-30

**Authors:** Sotirios Fragkostefanakis, Dimitrios Kaloudas, Panagiotis Kalaitzis

**Affiliations:** ^1^Molecular Cell Biology of Plants, Department of Biosciences, Goethe University, Frankfurt, Germany; ^2^Horticultural Genetics, Department of Horticultural Genetics and Biotechnology, Mediterranean Agronomic Institute of Chania, Chania, Greece

**Keywords:** PDCA, Prolyl 4 hydroxylases, Arabinogalactan-proteins, roots, hypocotyls, hydroxyproline, *Solanum lycopersicum*

## Abstract

Pyridine 2,4-dicarboxylic acid is a structural analog of 2-oxoglutarate and is known to inhibit 2-oxoglutare-dependent dioxygenases. The effect of this inhibitor in tomato seedlings grown in MS media supplied with various concentrations of PDCA was investigated, resulting in shorter roots and hypocotyls in a dose-dependent manner. The partial inhibition of growth in roots was more drastic compared to hypocotyls and was attributed to a decrease in the elongation of root and hypocotyl cells. Concentrations of 100 and 250 μM of PDCA decreased hydroxyproline content in roots while only the 250 μM treatment reduced the hydroxyproline content in shoots. Seedlings treated with 100 μM PDCA exhibited enhanced growth of hypocotyl and cotyledon cells and higher hydroxyproline content resulting in cotyledons with greater surface area. However, no alterations in hypocotyl length were observed. Prolyl 4 hydroxylases (P4Hs) are involved in the O-glycosylation of AGPs and were also highly expressed during seedling growth. Moreover PDCA induced a decrease in the accumulation of HRGPs and particularly in AGPs-bound epitopes in a dose dependent-manner while more drastic reduction were observed in roots compared to shoots. In addition, bulged root epidermal cells were observed at the high concentration of 250 μM which is characteristic of root tissues with glycosylation defects. These results indicate that PDCA induced pleiotropic effects during seedling growth while further studies are required to better investigate the physiological significance of this 2-oxoglutarate analog. This pharmacological approach might be used as a tool to better understand the physiological significance of HRGPs and probably P4Hs in various growth and developmental programs in plants.

## Introduction

The final plant organ size is controlled by environmental and genetic factors that coordinate cell expansion and division (Estevez et al., 2006; Gonzalez et al., 2010). The plant cell wall has a dominant role in cell growth through relaxation of the cross linking polymers, composition of new ones and rearrangement of its existing components (Tsukaya and Beemster, 2006) while cell growth is also regulated by hormones and mainly ethylene and the ethylene-auxin crosstalk (Hu et al., 2006). Hydroxyproline rich glycoproteins (HRGPs) are structural and functional components of cell wall and their participation in cell division and enlargement is well-known (Kieliszewski and Lamport, 1994; Nothnagel, 1997; Seifert and Roberts, 2007).

The 2-oxogulatarate dependent dioxygenase (2-ODDs) analogs comprising pyridine dicarboxylic acids, N-heterocycles and cyclohexanediones were extensively applied as competitive and non-competitive inhibitors of 2-ODDs (Xu et al., 2008). Among them pyridine 2,4-dicarboxylic acid (PDCA) suppressed ethylene production in carnation flowers and as a result delayed flower senescence (Vlad et al., 2010). In addition, it prolonged the shelf-life of a wide range of spray cut carnation flowers by enhancing flower opening in addition to retardation of senescence (Satoh et al., 2013, 2016; Sugiyama and Satoh, 2015).

Moreover, the induction of ethylene production by mechanical wounding was suppressed by PDCA in pericarp discs of mature green tomatoes due to either lack of induction and/or suppression of ethylene biosynthetic genes (Fragkostefanakis et al., 2012). PDCA also inhibited the *in vitro* enzyme activity of ACC oxidase (Fragkostefanakis et al., 2012) and recombinant P4Hs in carnation (Vlad et al., 2010), Arabidopsis (Tiainen et al., 2005), *Chlamydomonas reinhardtii* and *Volvox carteri* green algae (Kaska et al., 1987). PDCA down-regulated the climacteric ethylene in carnation flowers and inhibited the *in vitro* activity of DcP4H1 and DcP4H2 suggesting a putative correlation (Vlad et al., 2010).

Ethyl 3,4-dihydroxybenzoic acid (EDHB) is another structural analog of 2-oxoglutarate with similar to PDCA structure which was shown to inhibit P4H activity *in vitro* (Majamaa et al., 1986; Vlad et al., 2010). Moreover, the α,α-dipyridyl (DP) which chelates the cofactor ferrous (Fe^2+^) was reported to act as an inhibitor of *in vitro* P4H activity (Yuasa et al., 2005). Both P4H inhibitors were implicated in alterations in physiological programs in plants (Moriguchi et al., 2011; Velasquez et al., 2011, 2015).

Another inhibitor of P4H activity, the proline analog 3,4-dehydroproline (DHP), suppressed proline hydroxylation *in vivo* in carrot root slices causing an altered HRGP (Cooper and Varner, 1983) while proline rich proteins were not detectable in soybean cells treated with DHP (Schmidt et al., 1991). Moreover, exposure of Arabidopsis to DHP enhanced cell elongation indicating that HRGPs might be required to prevent the final length of root cells (De Cnodder et al., 2005).

Arabidopsis T-DNA knock out mutants of AtAGP19 exhibited growth-related phenotypes such as a delay in growth with shorter hypocotyls and inflorescence stems as well as smaller rosette leaves due to alterations in cell division and expansion (Yang et al., 2007). Moreover, the T-DNA knock out mutants of two Arabidopsis hydroxyproline galactosyltransferases, GALT5 and GALT2, displayed an inhibition of root growth and shorter root hairs of lower density especially in the double mutant (Basu et al., 2015) while the seedlings of the Arabidopsis *sos5* mutant exhibited shorter roots under saline conditions (Shi et al., 2003). Over expression of AGPs such as the *Cucumis sativus* CsAGP1 in tobacco resulted in promotion of stem growth (Park et al., 2003) while the over expression of Le-AGP1 in tomato plants enhanced lateral shoot growth and reduced elongation of the central stem (Sun et al., 2004).

HRGPs are hydroxylated by P4Hs and subsequently are O-glycosylated. Therefore, their physiological significance can be exploited using inhibitors of peptidyl prolyl hydroxylation (Cooper and Varner, 1983), since inhibition of hydroxyproline formation indirectly disrupts O-glycosylation and blocks the attachment of the carbohydrate side chains which partially determine their function (Zhang et al., 2008).

In this report the application of PDCA in tomato seedlings resulted in phenotypes of shorter roots and hypocotyls due to alteration in cell size which were accompanied by a reduction of hydroxyproline content and a decrease in the content of AGP-bound epitopes.

## Materials and methods

### Plant material and treatment

Two hundred tomato seeds (*Solanum lycopersicum* “Ailsa Craig”) were disinfected using 100% ethanol for 10 sec and positioned on solid MS substrate (Murashige and Skoog, 1962) (Sigma, M5519) supplemented with 10 g/L sucrose and 0.8% agar. The petri dishes were placed vertically in a plant growth chamber for 10 days at 24°C temperature and photoperiod of 14 h light/10 h dark. PDCA (*P63395*; Sigma-Aldrich, St. Louis, MO) was dissolved in ddH_2_O sterilized and added at the appropriate concentrations (0–100–250 μM) in the MS. The root and hypocotyl length of a total of 30 seedlings was determined and the experiment was repeated 3 times.

### RNA extraction and cDNA synthesis

Total RNA was extracted from pericarp discs according to Bachem et al. (1998) and reverse-transcribed with SuperScript™ II RNase H^−^ Reverse Transcriptase (Invitrogen, Carlsbad, CA) while Oligo dT_12−18_ primers were used for cDNA synthesis.

### Quantitative real time PCR analysis

Gene expression of 9 putative tomato P4Hs was quantified by using real-time PCR as described previously (Fragkostefanakis et al., 2012). Tomato P4Hs were under the following unigenes accession numbers: *SlP4H1*: SGN-U580108; *SlP4H2*: SGN-U566162; *SlP4H3*: SGN-U571903; *SlP4H4*: SGN-U564386; *SlP4H5*: SGN-U566163; *SlP4H6*: SGN-U562958; *SlP4H7*: SGN-U578386; *SlP4H8*: SGN-U571904; *SlP4H9*: SGN-U569769. The actin cDNA was as internal standard. Three biological replicates were performed. The primers used for the qRT-PCR analysis are shown in Table S1.

### Extraction of hydroxyproline concentration

Hydroxyproline was extracted (Arrigoni et al., 1977) and determined by the calorimetric procedure of Firschein and Shill (1966). Seedlings were removed from the substrate, washed from the agar and roots were separated from stems. The free amino acids in the tissues were removed by boiling with 80% ethanol for 15 min (4 times) followed by additional rinse with 100% ethanol for 15 min. Afterwards tissues were dried at 90°C and the dry weight was determined. The dried sample containing cytoplasmic and cell wall proteins hydrolyzed in 6N HCl solution at 110°C for 18 h, filtrated, concentrated and finally re-dissolved in ddH_2_O. The concentrating and dissolving steps repeated 3 times. The tissue content of hydroxyproline was determined by the calorimetric procedure (Firschein and Shill, 1966).

### Calorimetric determination of hydroxyproline

Samples of 3 ml of each hydroxyproline extract were mixed with 4 ml isopropanol, 1 ml of oxidase factor and incubated in room temperature. Erlich reagent of 10 ml was added to the mix, boiled for 2 min, chilled on ice and left in room temperature for 90 min. The optical density of the samples was determined at 575 nm via spectrophotometer (Hitachi, Model U-2001 UV/Visible Spectrophotometer). Blank didn't contain oxidase factor and reference curve was established with known hydroxyproline values (Sigma, H365G).

### Determination of root and hypocotyl epidermal cells

The effect of PDCA in root and hypocotyl epidermal cell growth was determined by microscopy of fully elongated cells in the area of 1–2 cm of cell elongation zone. Macgregor et al. (2008) method was followed to clear the roots and hypocotyl from the excess of agar. Once the roots were separated from stems, they were incubated sequentially for 15 min at 20% methanol which had been acidified with 4% HCl at 55°C and in 7% NaOH in 60% ethanol for another 15 min. The tissues were placed on slides and a glycerol droplet was added before covered with a glass. The observation was carried out under a light microscope (LeicaDM-LB). The area of 10 epidermal hypocotyl and 30 epidermal root cells from 10 seedlings was estimated with the software ProgRes® CapturePro 2.1.

### Determination of cotyledons area

Cotyledons were removed from seedlings, and placed in stereoscope equipped with a ProgResC12plus camera (JENOPTIK) and their area was determined according to Fragkostefanakis et al. (2014).

### Protein analysis

#### Protein extraction and total protein estimation

Protein extraction was performed according to Woodson (1987) with few modifications as described by Fragkostefanakis et al. (2014). Total protein concentration was determined by the Bradford method (Bradford, 1976).

#### SDS-PAGE

The SDS-PAGE was performed according to Sambrook et al. (1982). Protein extract (10 μg) samples were run at a constant current of 100 V through the stacking gel, and then the current was increased to 150 V. Following SDS-PAGE, the gel was placed facing a layer of PVDF membrane pre-moistened with 100% methanol. The transfer took place at 60 V for 2 h at 4°C.

#### Western blot analysis

Western blot analysis was performed as described previously (Fragkostefanakis et al., 2012). The primary antibodies Jim8 (Pennell et al., 1991) and Jim13 (Knox, 1997) were granted from Paul Knox Cell Wall Lab (University of Leeds, UK).

### Statistical analysis

Analysis of variance (ANOVA) was performed using the SPSS for Windows (version 17.0.0) and means were separated by Duncan's multiple range test at a 0.05 level.

## Results

### Seedling growth and hydroxyproline concentration

**Figure 1:**
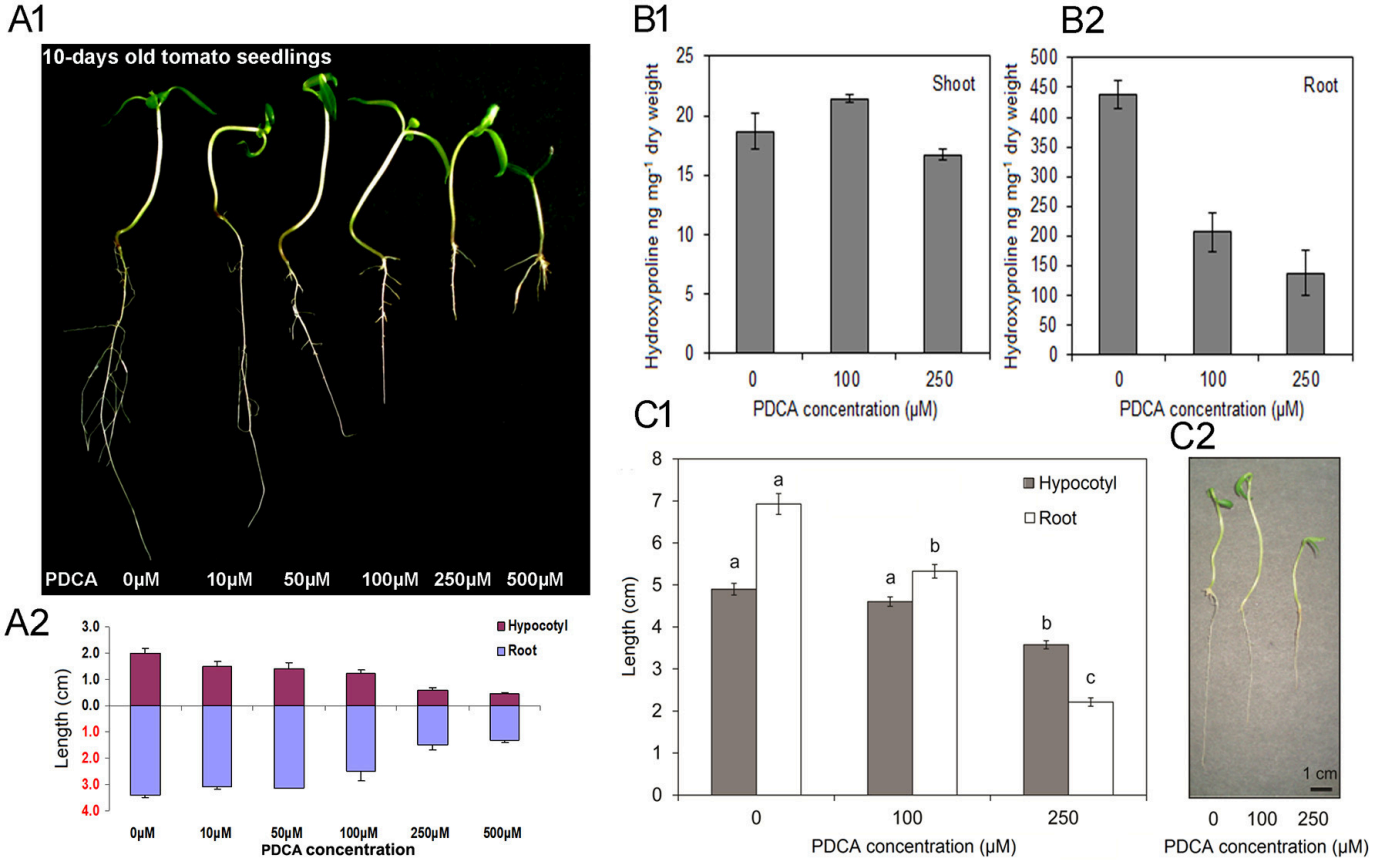
The effect of various PDCA concentrations on 10-days old tomato seedlings. **(A1)** Seedlings grown in 0, 10, 50, 100, 250, and 500 μM PDCA. **(A2)** Hypocotyl and root length of the PDCA-treated seedlings. **(B1)** Hydroxyproline content of the shoots and **(B2)** the roots, of seedlings grown in 0, 100 and 250 μM PDCA. **(C1)** Hypocotyl and root length of seedlings grown in 0, 100, and 250 μM PDCA. **(C2)** Representative seedlings grown for 10 days in 0, 100, and 250 μM. Each experiment represented the average of 3 biological replicates with the standard errors and each replicate was comprised of 30 seedlings. Mean values accompanied by a different letter indicate statistically significant differences according to Duncan new multiple range test at a 0.05 level.

The effect of PDCA on tomato seedling growth was investigated with five different concentrations of 10, 50, 100, 250, and 500 μM. Seed germination was not affected by the presence of the PDCA on the medium, as more than 90% of the seeds had sprouted up on the fifth day of the experiment. Concentrations below 100 μM did not alter the seedlings growth (Figures 1A1,A2) while the highest concentration of 500 μM induced toxic effects on seedlings by causing browning of the roots (Figure 1A1). Therefore, two concentrations of 100 and 250 μM were selected for further studies. Initially the hydroxyproline content in shoots and roots of 100 and 250 μM PDCA-treated seedlings was determined considering the inhibitory effect of 2-oxoglutarate analog on the activity of P4Hs in various plant species. Higher hydroxyproline content of almost 20-fold was determined in the roots (Figure 1B2) compared to the shoots (Figure 1B1). PDCA reduced the hydroxyproline levels in the roots by 50% and by more than 70% at concentrations of 100 and 250 μM, respectively (Figure 1B2). However, minor, non-significant changes were observed in the shoots of PDCA-treated seedlings (Figure 1B1). The hypocotyl and root length was also determined after 10 days of growth (Figure 1C1). The hypocotyl length was decreased by 1.5 cm at 250 μM of PDCA while no significant changes were observed at 100 μM (Figure 1C1). Shorter roots by 5 and 1.7 cm were observed at 250 and 100 μM, respectively indicating more drastic changes in the length of roots compared to hypocotyls (Figure 1C2).

### Expression analysis of putative tomato P4Hs during seedling growth

**Figure 2:**
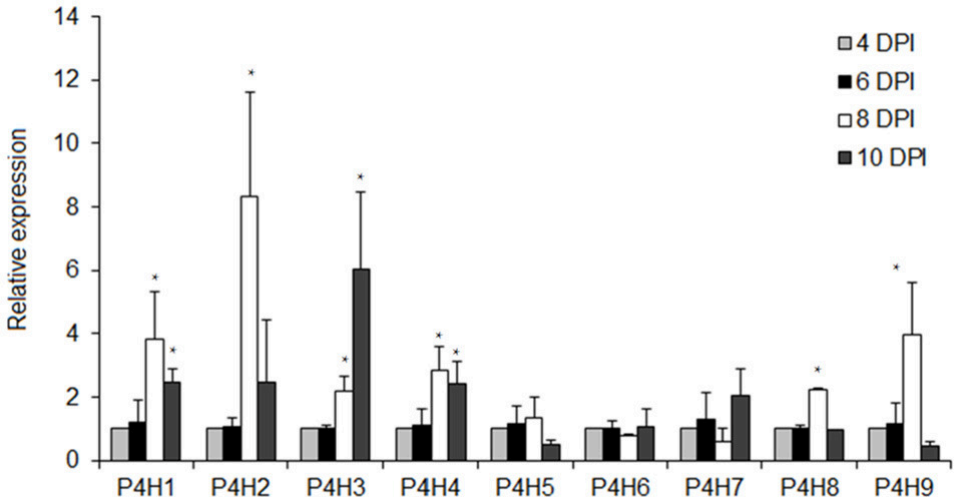
Expression analysis of nine tomato prolyl 4 hydroxylases in different tomato seedling stages of 4, 6, 8, and 10 DPI (Days Post Imbibition). Relative expression was calculated based on the comparative Ct method using actin as internal standard. The Ct value for each gene was normalized to the Ct value for actin and was calculated relative to a calibrator (4 DPI) using the formula 2^−DDCt^. Vertical bars are the average S.E. of three biological replicates. The ^*^indicates significant difference in the gene's relative expression.

The transcript levels of nine putative P4Hs were determined in tomato seedlings considering that PDCA is a potent inhibitor of P4H activity *in vitro* in several plant species. Six of them, P4H1, P4H2, P4H3, P4H4, P4H8, and P4H9 showed significant increase in expression after 8 DPI ranging from 8-fold for P4H2, 4-fold for P4H1 and P4H9 and 2-fold for P4H3, P4H4 and P4H8 (Figure 2). Only the transcript levels of P4H6 increased by 6-fold after 10 DPI (Figure 2). Among them, four exhibited a gradual increase in expression which was initiated mainly after 8 and 10 days post-imbibition (DPI) (Figure 2). The highest transcript abundance was observed in P4H2 followed by P4H3, P4H1, and finally P4H9 (Figure 2). The rest of the genes showed minimal up-regulation mainly after 8 and 10 DPI (Figure 2). These results indicate up-regulation of several putative P4Hs during tomato seedling growth.

### Determination of the root and hypocotyl cell area

**Figure 3:**
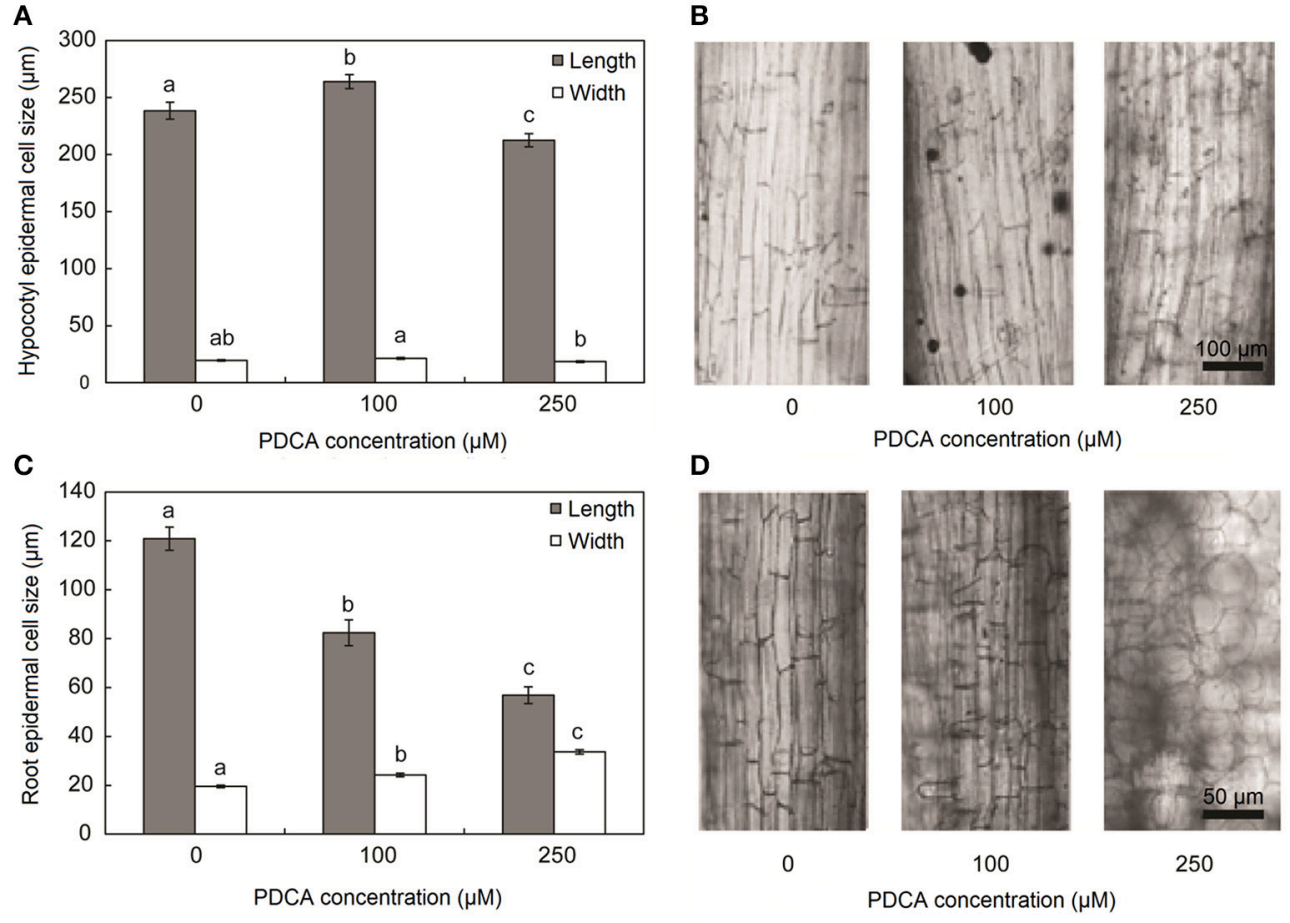
Hypocotyl and root cell size of seedlings grown for 10 days in MS media supplied with PDCA. **(A)** Length and width of hypocotyl epidermal cells grown in 0, 100, and 250 μM. **(B)** Longitudinal sections of fully elongated hypocotyl cells grown in 0, 100, and 250 μM. **(C)** Length and width of root epidermal cells grown in 0, 100, and 250 μM. **(D)** Longitudinal sections of fully elongated root cells grown in 0, 100, and 250 μM. Mean values accompanied by a different letter indicate statistically significant differences according to Duncan new multiple range test at a 0.05 level.

**Figure 4:**
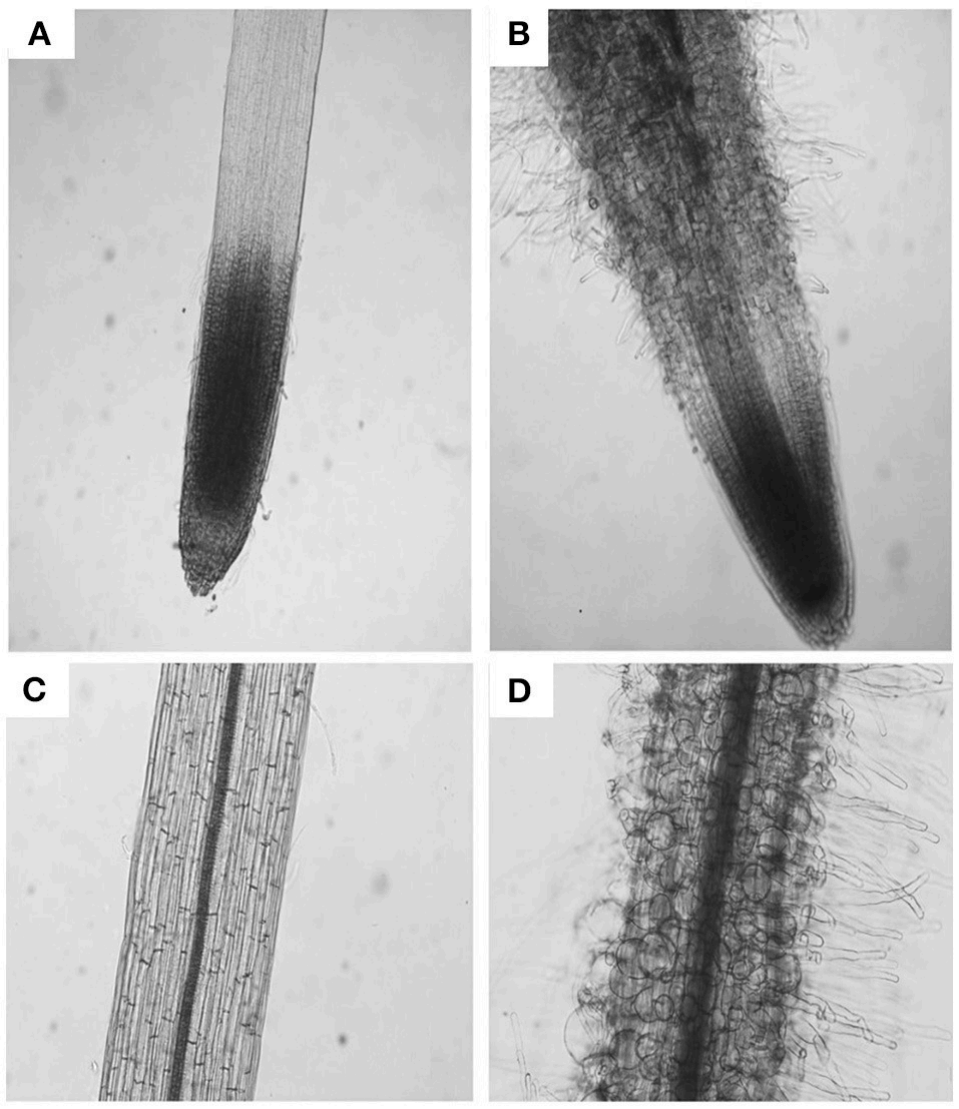
The effect of PDCA on the morphology of the root cells in 10-day old tomato seedlings. Root and root cell shape and morphology of 10 day-old seedlings after growth on MS media supplied with 0 **(A,C)** or 250 μM PDCA **(B,D)**.

The parameters of length and width of fully elongated cells in hypocotyls and roots were determined in response to PDCA. The length of the hypocotyl cells was higher at 100 μM and lower at 250 μM compared to the control (Figures 3A,B). The length of the hypocotyl was around 260 and 210 μm after treatment with 100 μM and 250 μM PDCA, respectively. This is a significant difference of 50 μm in length (Figure 3A). The same cells at 100 and 250 μM were of similar width compared to the control but of different width with each other with wider the cells of 100 μM (Figures 3A,B). The epidermal cells of the root were shorter and wider compared to the control in a dose dependent manner considering that the cells exposed to 250 μM were shorter compared to the control and wider than those exposed to 100 μM (Figures 3C,D). The length of the 250 μM root epidermal cells was around 60 μm; half the length compared to the control and 20 μm shorter compared to the 100 μM PDCA (Figure 3C). Moreover, the 250 μM-treated cells had a distinctly flattened form with round, swollen, bulged shape (Figure 4). The number of bulged cells was associated to the concentration of PDCA but their presence was not constant (Figure 4).

### 100 μM PDCA concentration affects seedlings cell number and cotyledons size

**Figure 5:**
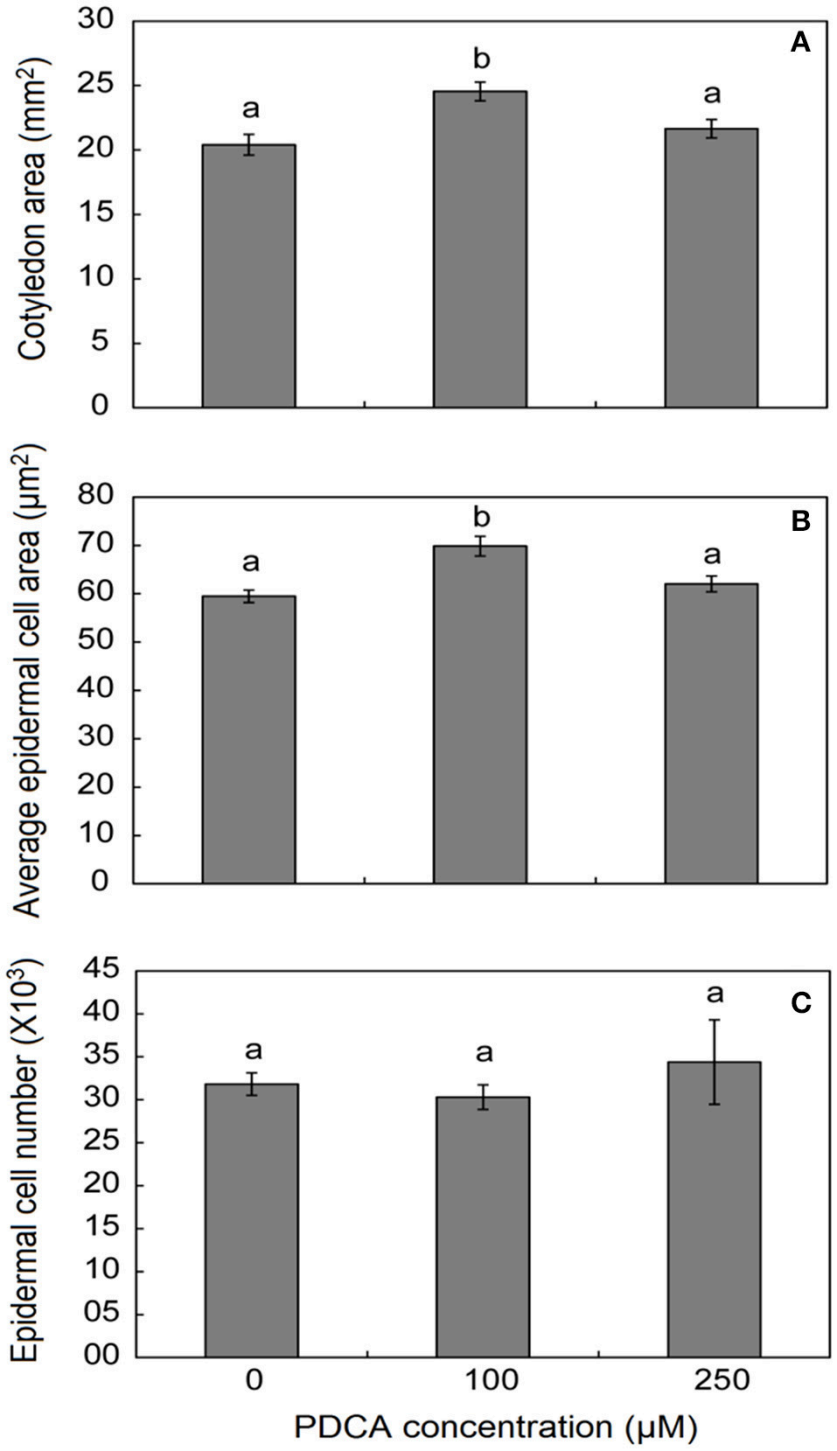
The effect of PDCA on the cotyledon and epidermal cell area and cell number in 10-day old tomato seedlings. **(A)** The cotyledon area of seedlings treated with 0, 100, and 250 μM. **(B)** The average epidermal cell area of cotyledons in seedlings treated with 0, 100, and 250 μM. **(C)** The average epidermal cell number in seedlings treated with 0, 100, and 250 μM. Mean values accompanied by a different letter indicate statistically significant differences according to Duncan new multiple range test at a 0.05 level. Each experiment represented the average of 3 biological replicates with the standard errors. In each replicate the area of 10 cotyledons was determined as well as the area of 15–20 epidermal cells per cotyledon.

The surface area of cotyledons in young seedlings was larger only when grown in 100 μM PDCA and not in 250 μM indicating alterations in the response under different PDCA concentrations (Figure 5). In addition, the average cell area was also larger only in 100 μM treated cotyledons and not in 250 μM while the average cell number was similar in treated and un-treated hypocotyls (Figure 5).

### Determination of AGPs content in PDCA-treated seedlings

**Figure 6:**
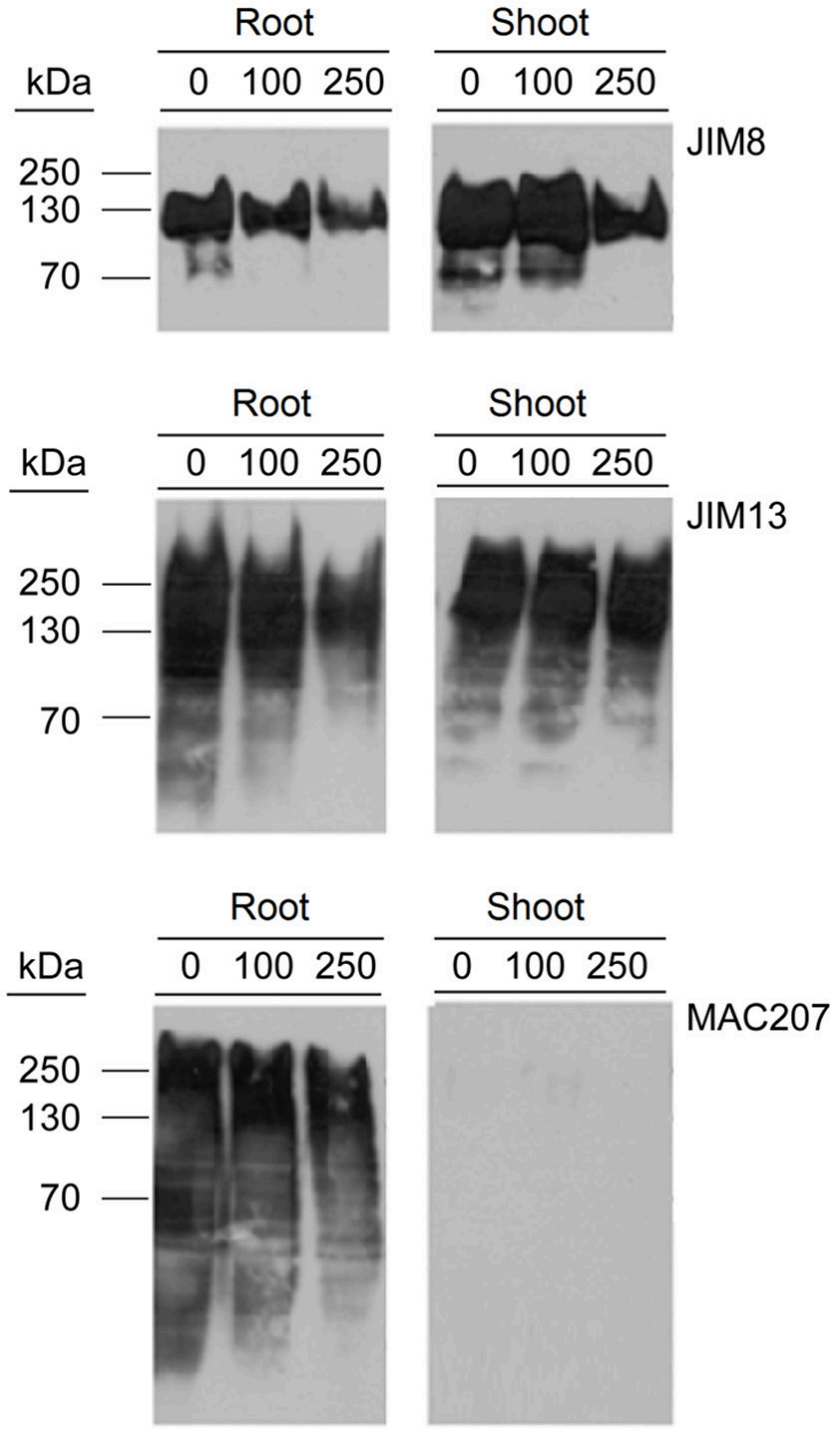
The effect of PDCA on the expression of AGPs in 10-day old tomato seedlings using western blot analysis. Tomato seedlings were treated with 0, 100, and 250 μM and total proteins were extracted from the roots and shoots for western blot analysis by using JIM8, JIM13, and MAC207 antigens. Loading per lane was 10 μg of total proteins.

Hydroxyprolines in nascent polypeptides are necessary for the synthesis of functional glycoproteins. Therefore, the effect of PDCA in the content of AGP proteins was determined by using western blot analysis with the monoclonal antibodies JIM8, JIM13, and MAC207 which recognize different AGP carbohydrate epitopes. The molecular weight of JIM8- and JIM13-bound epitopes ranged between 70–150 kDa and 30–300 kDa, respectively (Figure 6). PDCA reduced the content of JIM8-, JIM13-, and MAC207-bound epitopes in the roots in a dose-dependent manner with the lower content of AGPs being observed at 250 μM (Figure 6). In the shoot of young seedlings, only the JIM8-bound epitopes showed a lower content at 250 μM PDCA while no changes were observed in JIM13-bound epitopes (Figure 6). However, the MAC207 antibody did not cross-reacted with any AGP protein in the shoots of young seedlings (Figure 6).

## Discussion

PDCA, a 2-oxoglutarate analog, decreased the root and hypocotyl length of young tomato seedlings in a dose-dependent manner. The inhibition of growth in roots was more drastic compared to hypocotyls and was observed in two concentrations of PDCA, 100 and 250 μM. These alterations in length could be attributed to a decrease in the elongation of root and hypocotyl cells and was accompanied by a decrease in hydroxyproline content. These results are in accordance with previous studies using the analog of proline, 3,4-dehydroproline DHP, in tomato seedling roots (Bucher et al., 1997) and green beans (Fowden, 1963). Although the treatment of seedlings with 100 μM PDCA did not significantly affect the length of hypocotyls, it enhanced the growth of epidermal cells of both hypocotyls and cotyledons and their hydroxyproline content, thus developing cotyledons with greater surface area suggesting a putative relation between hydroxyproline content, cell length and cell area in hypocotyls and cotyledons, respectively. Promotion of cell growth has been observed in Arabidopsis and onion roots after a brief treatment with DHP (De Tullio et al., 1999; De Cnodder et al., 2005), however, the effect of the inhibitor on the above ground part of the plants was not tested in these studies. The fact that different concentrations of PDCA cause opposite phenotypes in different tissues of tomato seedlings such as cotyledons, hypocotyls and roots might be attributed to more than one mode of actions of this analog in various physiological programs of seedling growth.

In Arabidopsis, an association between root hair growth and proline hydroxylation was initially demonstrated with the inhibition of root hair elongation by using EDHB and DHP (Velasquez et al., 2012). Similar inhibition was observed in the T-DNA knock out mutants of three P4Hs, AtP4H2, AtP4H5, and AtP4H13, leading also to shorter root hair due to the inhibition of O-glycosylation in HRGPs such as extensins suggesting involvement in root hair tip growth (Velasquez et al., 2012, 2015). Moreover, application of DHP in tobacco plantlets resulted in reduction of Arabinogalactan-type O-glycosylation possibly through inhibition of proline hydroxylation (Moriguchi et al., 2011). Therefore, the use of inhibitors of proline hydroxylation might be an efficient way to determine the physiological significance of HRGPs (Cooper and Varner, 1983).

The presence of swollen, bulged epidermal cells was observed in the roots of seedlings exposed to 250 μM of PDCA (Figures 3, 4). This phenotype was previously observed in the trichoblast defective Arabidopsis mutant for the biosynthesis of galactose *reb1-1* (Seifert et al., 2002) and at the epidermal root cells, which were grown in nutrient medium in the presence of β-Yariν (Willats and Knox, 1996). The trichoblast of the *reb1-1* mutants have lower concentration of AGPs (Andeme-Onzighi et al., 2002) and lower content of xyloglucan (Nguema-Ona et al., 2007). The β-Yariν is known to bind AGPs and disrupts their function. Therefore, the occurrence of a similar phenotype in PDCA-treated seedlings might be attributed to partial inhibition of O-glycosylation in AGPs due to a reduction in proline hydroxylation. This might explain the decrease in theJIM8-, JIM13-, and MAC207-bound epitopes.

Furthermore, it was observed that the root epidermal cells were shorter in length and wider in width which is due to the lower concentration of AGPs, as glycoproteins are necessary for the anisotropic growth of the cells and for the orientation of the microtubules (Andeme-Onzighi et al., 2002). It was recently reported that double mutants of Arabidopsis AGP glycosyltransferases, *galt2* and *galt5*, showed shorter roots, shorter inflorescences as well as several other phenotypes (Basu et al., 2015). In roots the β-Yariv inhibits root elongation; an inhibition which is attenuated in the double mutants indicating a role for the AG polysaccharides in root elongation (Basu et al., 2015). However, single mutants did not show any phenotype indicating that certain levels of glycosylated AGPs are necessary for certain physiological programs (Seifert et al., 2002).

It was suggested long time ago that ethylene might control growth of plant cells by regulating the hydroxylation of cell wall proteins (Ridge and Osborne, 1971). Specifically, it was shown that hydroxyproline rich cell wall proteins were necessary in preventing cell wall extension when growth expansion ceased (Sadava et al., 1973). PDCA might regulate hydroxyproline content through regulation of ethylene production considering that in *pisum sativum* hydroxyproline and peroxidases in cell walls were regulated by ethylene (Ridge and Osborne, 1970).

Inhibitors such as PDCA, a structural analog of 2-oxoglutarate, might affect the activity of other 2-ODD enzymes such as the flavonoid 3′-hydroxylase, flavonol synthase and the gibberellin 3β-hydroxylase (Britsch and Grisebach, 1986; Brown et al., 1998; Halbwirth et al., 2006). However, it was recently reported that gibberellin is not associated with the acceleration of carnation flower opening by PDCA in spray-type cultivars (Morita et al., 2017).

Further investigation on the mode of action of PDCA is required by high throughput approaches such as proteomics, metabolomics and transcriptomics in order to elucidate its physiological significance in plant growth and development.

## Author contributions

PK and SF: designed the experiments; SF: performed the experiments and PK, SF, and DK drafted the manuscript.

### Conflict of interest statement

The authors declare that the research was conducted in the absence of any commercial or financial relationships that could be construed as a potential conflict of interest.
