# Supplementary material for: Pyridine 2,4-Dicarboxylic Acid Suppresses Tomato Seedling Growth
Source: Front Chem. 2018 Jan 30;6:3. doi: 10.3389/fchem.2018.00003 (PMC5797587; doi:10.3389/fchem.2018.00003)
Supplement: Supplementary file 1 [file Table1.DOCX]

**Table S1.** Genes and oligonucleotides used in real time PCR experiment

| Gene | Unigene | Primer name | Primer sequence | Fragment length (bp) | Primer concentration |
| --- | --- | --- | --- | --- | --- |
| *SlP4H1* | SGN-U580108 | SlP4H1RT_f | 5´-CGAGTAAATGCCTGTACAATA-3´ | 146 | 300 nM |
|  |  | SlP4H1RT_r | 5´-TCGGAGAGATGGAAAATGAAG-3´ |  | 300 nM |
| *SlP4H2* | SGN-U566162 | SlP4H2RT_f | 5´-CGAGAGTCGAGACAGTGACAGTAA-3´ | 145 | 300 nM |
|  |  | SlP4H2RT_r | 5´-GCATGCTTGCACCTGTATGTGGA-3´ |  | 300 nM |
| *SlP4H3* | SGN-U571903 | SlP4H3RT_f | 5´-GAATAGAGCCACCACACATGAA-3´ | 144 | 300 nM |
|  |  | SlP4H3RT_r | 5´-TCGAATGAAGCATCTCACATCTT-3´ |  | 300 nM |
| *SlP4H4* | SGN-U564386 | SlP4H4RT_f | 5´-CAATGGACCGTCAACTTCCTT-3´ | 151 | 300 nM |
|  |  | SlP4H4RT_r | 5´-CTACCAGTCACCAATTTCAGCA-3´ |  | 300 nM |
| *SlP4H5* | SGN-U566163 | SlP4H5RT_f | 5´-GTTAACTTGTTTCTTCCACTTGA-3´ | 147 | 300 nM |
|  |  | SlP4H5RT_r | 5´-AGTACAAGATTACTTGCAAAATTGAG-3´ |  | 900 nM |
| *SlP4H6* | SGN-U562958 | SlP4H6RT_f | 5´-CAAGGAAGAATGTGAATACTTGATAA-3´ | 105 | 300 nM |
|  |  | SlP4H6RT_r | 5´-GTACGAACCCTGCTATCTTTACT-3´ |  | 900 nM |
| *SlP4H7* | SGN-U578386 | SlP4H7RT_f | 5´-GTAGCCACTCACTGGAACCTT-3´ | 119 | 300 nM |
|  |  | SlP4H7RT_r | 5´-GTAATGGGAGCAACCAAGACTCA-3´ |  | 300 nM |
| *SlP4H8* | SGN-U571904 | SlP4H8RT_f | 5´-CAGCTTTCAACCTCAAGCC-3´ | 137 | 300 nM |
|  |  | SlP4H8RT_r | 5´-GTGCCGAGTGGATTTACTTTAC-3´ |  | 300 nM |
| *SlP4H9* | SGN-U569769 | SlP4H9RT_f | 5´-CTATGGCTGGCACTGGTAAAT-3´ | 199 | 300 nM |
|  |  | SlP4H9RT_r | 5´-ACAGTTCTTTGCCTCATCCA-3´ |  | 300 nM |
| *ACTIN* | SGN-U580609 | ActinRT_f | 5´-GTCCCTATTTACGAGGGTTATGCT-3´ | 127 | 300 nM |
|  |  | ActinRT_r | 5´-GTTCAGCAGTGGTGGTGAACA-3´ |  | 300 nM |
